# Group size and labour demands determine division of labour as a consequence of demographic stochasticity

**DOI:** 10.1098/rstb.2024.0206

**Published:** 2025-03-20

**Authors:** Christoph Netz, Tim W. Fawcett, Andrew D. Higginson, Michael Taborsky, Barbara Taborsky

**Affiliations:** ^1^ Behavioural Ecology Division, Institute of Ecology and Evolution, University of Bern, Bern, Switzerland; ^2^ Centre for Research in Animal Behaviour (CRAB), University of Exeter, Exeter, UK

**Keywords:** division of labour, task specialization, learning, group size

## Abstract

Division of labour (DoL) is most prominently observed in eusocial insects but also occurs in much smaller cooperative groups where all individuals could potentially perform any task. In such groups, previous experience and learning are the most important mechanisms underlying specialization. Using behavioural simulations, we investigate the dynamics of task specialization in groups of various sizes and with different constraints on the choice of task. We assume that individuals choose tasks by weighing their own competence to perform a task against the group requirement of how much that task needs to be performed. We find that task specialization occurs even if individuals choose tasks based solely on the group’s needs rather than their own competence. As large groups are less affected by demographic stochasticity, they can more accurately distribute labour across tasks, and individuals become more effective due to a reduced need to switch between tasks. This effect is enhanced if groups must perform a larger number of tasks. However, from an evolutionary point of view, individuals in larger groups develop a greater responsiveness to group requirements than those in small groups when labour variation carries a fitness penalty and thus will more readily switch between tasks. Small groups thus seem less able to distribute labour optimally over tasks through increased switching, and therefore evolve to ignore task imbalances up to a higher level before the threshold to switch between tasks is crossed. Further, we find that selection on learning ability is stronger in small than in large groups. We conclude that the reason why DoL may emerge more readily in large groups might not be due to a group-size effect on optimal decision-making, but rather because of a lower degree of variation of the labour distribution as a consequence of demographic stochasticity.

This article is part of the theme issue ‘Division of labour as key driver of social evolution’.

## 1. Introduction

Division of labour (DoL), the uneven distribution of tasks among the members of a cooperative group, is a widespread natural phenomenon in social animals. DoL occurs most conspicuously in eusocial insects [1] but is also seen in a range of other group-living organisms such as other arthropods, birds, mammals and even bacteria [2–9]. Understanding DoL is of great interest not only because of its pervasive influence on the lives of our own species [10,11], but also because it constitutes a crucial step in the recurring evolutionary transitions of group formation, functional differentiation of its members and integration towards higher levels of organization [12,13].

The forms that DoL can take vary widely across the tree of life. The most extreme form of DoL are the sterile, morphologically differentiated castes found in some eusocial insect species, in which individuals are specialized for functions such as reproduction, defence or foraging that are pursued for their entire lives [14]. Although even individuals belonging to specialized morphs can often still perform a number of different tasks, such levels of specialization come at a large opportunity cost [15,16].

Another form of DoL is temporal polyethism, where individuals specialize into different tasks in accordance with their age [17]. Examples can again be found among the eusocial insects, such as the succession of tasks performed by honeybees (*Apis mellifera*) but also in group of vertebrates that form hierarchies based on size and age. In the social cichlid *Neolamprologus pulcher*, depending on circumstances, large group members may specialize in digging out the breeding shelter, while small individuals specialize in the protection of eggs [18]. In contrast to caste-based DoL, temporal polyethism is more plastic, with individuals being able to respond to group needs in switching between tasks [17].

DoL can also be spontaneous, in that individuals specialize on different tasks regardless of caste or age. Such DoL may take advantage of prior individual variation but more importantly arises through the general tendency of animals to develop habits and learn from experience [19,20]. The ubiquity of this mechanism allows DoL to arise spontaneously even in solitary species when experimentally grouped together [21,22]. Spontaneous specialization and learning can also play an important role in DoL in many eusocial insects [20], and indeed eusocial insects such as bees stand out for their exceptional learning abilities [23,24]. The plasticity of learned behaviour, however, also means that this form of DoL can be ephemeral and rather inconspicuous.

Group size has a crucial influence on the emergence of DoL [25]. Superficially, caste specialization and temporal polyethism seem to be largely limited to eusocial insects, especially those termites, bees and ants that form huge colonies with tens of thousands of individuals, whereas spontaneous DoL occurs in small groups of vertebrates as well. DoL more readily emerges when group size is experimentally increased [26–29]. Several theoretical studies have predicted that increased group sizes can promote the emergence of DoL [29–33]. Cooper & West [13] argued that group size can enhance DoL if the efficiency benefits of specialization increase with group size. Ulrich *et al*. [29] showed that task specialization increases with group size in a simple fixed-threshold model, due to increased homeostasis and reduced stochasticity in individual task choice. Nakahashi & Feldman [33] predicted an increase of DoL with increasing group size due to what effectively amounts to resource competition between group members, while Pacala *et al*. [30] and Gautrais *et al*. [31] predicted that group size will increase DoL due to enhanced uptake of information and interaction between individuals.

The most widely employed approach to modelling DoL is the response-threshold model, which assumes that individuals perform tasks if a certain stimulus threshold is surpassed [31,32,34–36]. Between-individual variation in this threshold due to genetic variation or phenotypic plasticity can then give rise to DoL [37], as long as within-individual variation is not overwhelming [38]. Response-threshold models often incorporate a feedback mechanism, whereby the repeated execution of a task lowers the threshold and thereby allows individual specialization to arise even in the initial absence of between-individual variation [35]. However, this widely modelled mechanism (cf. [39]) does not incorporate any improvement in task performance due to experience or learning. It seems plausible that individuals prefer to perform tasks that they are good at, while at the same time acting to reduce the group’s collective labour demands across the various tasks. Especially in smaller groups, where individuals can track the overall condition and needs of the group, the importance of the decision-making of the individual may have been under-emphasized.

To address this gap, in this study we investigate the relationship between group size and DoL using a novel model that highlights the role of learning and the relative importance of individual efficiency versus group labour requirements in spontaneous task specialization. We build an individual-based simulation model in which individuals cooperate to perform a number of tasks and improve their task competence through learning. Demographic stochasticity and the group requirement to work on multiple tasks may induce task switching. We use this model to assess how task switching and DoL depend on group size, the number of tasks and the rate at which individuals improve their task performance (e.g. through practice and learning).

## 2. Model description

We assume individuals live in a group and perform tasks that they select based on (i) their own ability depending on previous task experience and (ii) the requirements of the group for different tasks. By performing a task, individuals acquire experience and improve their task competence through learning. On the other hand, task switching may help to meet the requirements for each task in the face of demographic stochasticity.

### (a) Demography

We assume that time is split into discrete time steps, which might be an hour or a day. At each time step, individuals join the group with an average rate r. This can represent recruitment via both reproduction and immigration. For simplicity, we assume that individuals arrive in a naive state without prior task experience. Each time step, individuals die with probability d. Both arrival and death are implemented as stochastic processes, with the number of recruits drawn from a Poisson distribution P(r) and the number of deaths drawn from a binomial distribution B(N(t),d), where N(t) is the current group size. The groups are therefore subject to demographic stochasticity. Group sizes across consecutive time steps are determined by


(2.1) N(t+1)=B(N(t),d)+P(r)

The equilibrium group size is $N^{*}=r/d$, and the average life expectancy of individuals is 1/d (≈166 time steps for the results shown below). We considered three different recruitment rates (0.03, 0.06, 0.2), corresponding to small ($N^{*}=5$), intermediate ($N^{*}=10$) and large ($N^{*}=33$) groups. Parameters and default settings are listed in table 1.

**Table 1.** Parameters.

parameter	interpretation	values (default in bold)
r	recruitment rate	**0.03, 0.06, 0.2**
d	mortality	**0.006**
λ	task choice rate	**0.2**
K	number of tasks	**2, 3, 5**
f	responsiveness	**{0.0−1.0}**
μ	learning rate	0.01, 0.02, 0.05, **0.1**, 0.2
ν	forgetting rate	0.05, **0.1**
c	variation cost	**{0.0−5.0}**
variables		
G	work done *per capita*	—
σ	standard deviation of the standardized labour distribution	—

### (b) Learning and experience

The individuals perform K different tasks together as a group. Each individual i performs a single task $\tau_{i}\in \{1,K\}$ in each time step. Individuals gather experience E at a task k while it is performed and lose some of the benefit of this experience (referred to as ‘forgetting’) when performing another task


(2.2) $$E_{i,k}\left(t+1\right)=\left\{ \begin{array}{ll} \left(1-\mu \right)E_{i,k}\left(t\right)+\mu , & if\;k\;=\;\tau_{i}\\ \; & \;\\ \left(1-\nu \right)E_{i,k}\left(t\right), & otherwise \end{array}\right.$$

where μ and ν are the rates of learning and forgetting, respectively. Experience varies between 0 (the individual is naive to that task) and 1 (maximum experience). The acquisition of experience has diminishing returns, whereas the loss of experience follows an exponential decay. Experience is updated at each time step in all individuals of the group. We considered learning and forgetting rates of 0.1 per time step as the default setting in our simulations, and in addition investigated learning rates between 0.01 and 0.2 and an additional forgetting rate of 0.05 (table 1).

### (c) Task choice and competence

Individuals update their task on average every five time units, with intervals for each update drawn from an exponential distribution Exp(λ=0.2). We used a continuous time implementation, but a discrete time implementation would be equivalent. The competence p of individual i at task k is determined by the experience they have gathered so far. Naive individuals have a baseline competence of 1, and by gathering experience $E_{k}$ they can increase this competence up to a maximum of 1 additional unit. Task competence is therefore given by $p_{i,k}=1+E_{i,k}$.

The labour distribution L in the group is a vector with K elements that is the total amount of work done by the group in each task, which corresponds to the sum over individual competences in the task τ they performed.


(2.3) $$L_{k}=\sum_{i=1}^{N}\left\{ \begin{array}{ll} p_{i,k}, & if\;k=\tau_{i}\\ \; & \;\\ 0, & otherwise \end{array}\right.$$

Tasks are chosen based on an individual’s competence in a task relative to the other tasks it could perform, $p_{i,k}/\sum_{j=1}^{K}p_{i,j}$ and the distribution of the group’s labour $L_{k}/\sum_{j=1}^{K}L_{j}$ across all tasks:


(2.4) $$\tau_{i}=max\left[k\right]\left(\left(1-f\right)\frac{p_{i,k}}{\sum_{j=1}^{K}p_{i,j}}+f\left(1-\frac{L_{k}}{\sum_{j=1}^{K}L_{j}}\right)\right)$$

The parameter f (0≤f≤1) determines the extent to which individuals respond to the task requirements of the group, as opposed to their own competences for different tasks. For brevity, we will refer to *responsiveness*. If f=0, individuals choose tasks purely based on their own competence, ignoring group requirements. If f=1, individuals choose the task that is most needed in the group, irrespective of their own competence. Task choice is absolute, such that individuals always choose the highest scoring task in equation (2.4) no matter whether the score differences between tasks are large or small. When two or more tasks have the same score, e.g. in case of naive individuals without prior experience, individuals choose between these tasks at random.

As measures of group performance, we consider how much work is done overall and how evenly that work is distributed across tasks. Specifically, we calculated the mean work done *per capita*, G


(2.5) $$G=\frac{\sum L_{k}}{N}$$

and the standard deviation of the standardized labour distribution, σ


(2.6) $$\sigma =\sqrt{\sum_{k=1}^{K}{\left(\frac{L_{k}}{G}-\frac{1}{K}\right)}^{2}}$$

## 3. Results

Simulations were performed for a single group over an extended period of time. The group was initialized at equilibrium size $N^{*}=r/d$ without experience, followed by a burn-in period of 5000 time steps during which no data were recorded. We then ran simulations until the 10 millionth individual joined the group and recorded the average amount of work done per individual G and the variation of the labour distribution L as measured by its standard deviation σ. To record individual-level data such as number of task changes per lifetime or competence, we ran additional simulations over shorter time periods. In our main set of results, we ran simulations for three different average group sizes (5, 10, 33), either 2, 3 or 5 different tasks, and responsiveness from f=0 to 1 in increments of 0.01 (table 1). The life history of individuals was constant across these simulations, with an average life expectancy of 166 time steps and an update of task choice on average every five time steps. The group size dynamics are shown in electronic supplementary material, figure S1; whereas absolute variation in group size increases with group size, the coefficient of variation in group size decreases from small to large groups.

**Figure 1.**
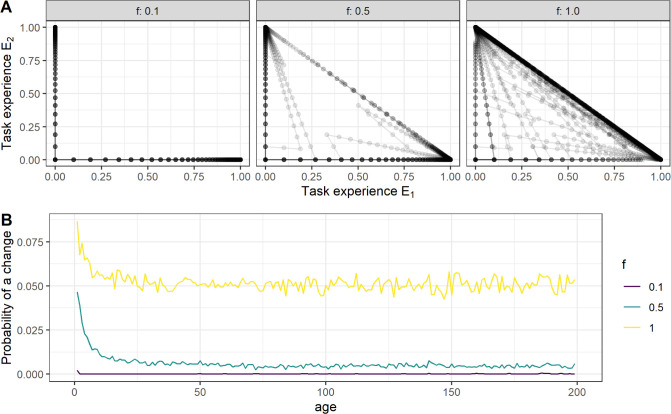
Experience and switching between tasks. (A) Experience across the lifetime of 100 randomly selected individuals, for two tasks (K=2) and three values of f(0.1, 0.5, 1.0), at average group size 10 (r=0.06). Individuals have a life expecancy of ca. 167 time steps, begin their life with zero experience ($E_{1}=E_{2}=0$) and from there gather experience in the task(s) they perform. Individuals that never switch tasks only accumulate experience in a single task, such that they gradually approach the maximum level of competence for that task. As responsiveness increases across the panels from left to right, individuals increasingly switch between tasks. At each time step, these individuals gather experience in one task and lose experience in the other. Data points show individuals at a certain time step, with consecutive time steps connected by lines. Each individual’s line and data points are shown with a transparency of 0.1, such that a point representing a single individual appears light grey, and 10+ overlapping individual data points appear black. (B) Task change frequency per time step in relation to individual age. As in (A), individuals hardly ever change between tasks at a responsiveness of f=0.1 but increasingly do so for responsiveness values of 0.5 and 1. Changes are made more frequently during early life when task experience is still low.

We found that for low values of f, individuals specialize on one task and never switch to another (figure 1A, left panel). As f increases, individuals begin to switch tasks more frequently. At f=1, individuals choose tasks purely based on the labour distribution in the group, seemingly corresponding to spontaneous DoL. Interestingly, even at f=1, we still find substantial task specialization: the average number of task changes per lifetime is below 1.5 across all parameter combinations (electronic supplementary material, figure S2), whereas random choice between two tasks every five time steps over a life expectancy of 166 time steps implies an expectation of 17 task changes. The low number of task changes occurs because individuals reach a somewhat stable distribution over tasks by choosing whichever task needs to be done during early life. Task changes occur subsequently only when this distribution is disturbed by mortality. Individuals are more likely to switch between tasks during early life, when they have yet to gather experience, than during later stages, when they have specialized on a particular task (figure 1B). Thus, we not only observe specialization of individuals into certain tasks but also a temporal polyethism, where young individuals switch tasks and thereby balance the group’s task needs, while older individuals persist in their specialized tasks for which they have gathered experience throughout life.

**Figure 2.**
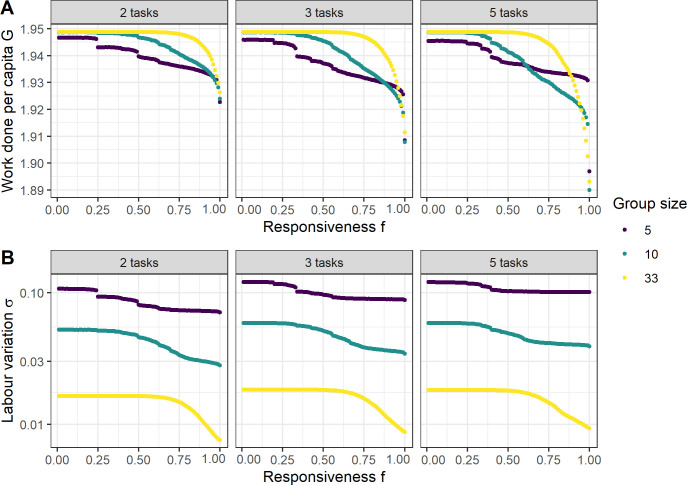
Task performance and variation. (A) Work done per capita in relation to responsiveness f and three different average group sizes (5, 10, 33), corresponding to recruitment rates of (0.03, 0.06, 0.2). (B) Variation between the work done in each task by the group, as measured by the standard deviation σ of the labour distribution. Each simulation was run until 50 000 individuals had been sampled. The work done *per capita* and labour standard deviation were averaged over all time steps.

These task choice dynamics affect the work done by groups and the variation observed in the labour distribution. As f increases, individuals are more likely to switch to tasks that are under-supplied at the group level thereby reducing the variation of the labour distribution (figure 2B). As individuals switch to tasks in which they have no previous experience, this temporarily reduces competence at the performed task and so the amount of work done decreases (figure 2A). The variation of the labour distribution is consistently greater in small groups than in large groups (compare lines in figure 2B). The work done in small groups starts to decrease at lower values of f than in medium or large groups, as labour variation is high and induces more frequent task switches. With larger numbers of tasks, the opposite effect occurs: whereas all the individuals in a large group start switching due to small variations between tasks, individuals in small groups are more likely to reach a stable distribution over tasks where no individual can reduce variation by switching (compare figure 2B, right panel, group sizes 5 and 33). The discrete drops in total work and variation around f values of 0.25 and 0.5 are due to the task choice decisions made in particular situations that occur frequently in small groups (e.g. if task 2 is left completely unattended, an individual exclusively specialized on the other task (competence $p_{1}=2$ versus $p_{2}=1$) will switch over according to equation (2.2) only if f>0.25).

**Figure 3.**
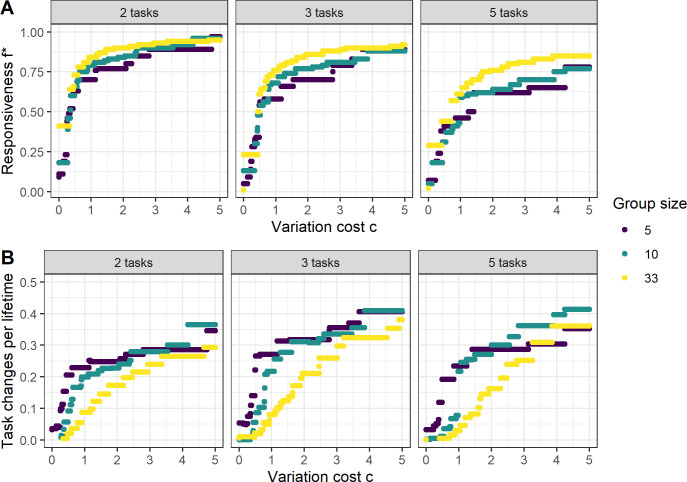
Optimal responsiveness and resulting division of labour. (A) The optimal responsiveness $f^{*}$ at which fitness (equation (3.1)) is maximized, as a function of the imposed cost c penalizing unequal labour distribution across tasks. These values are quite noisy, especially for low values of f where work *per capita* (G) and variation (σ) are almost flat (see figure 2). However, we observe that $f^{*}$ is lower if groups have to perform more tasks at the same time (moving across the panels from left to right), and that for intermediate values of variation cost c it appears that small groups have a smaller optimal responsiveness than large groups. (B) The average number of task changes that an individual makes per lifetime in relation to variation cost c, using the optimal responsiveness $f^{*}$. Although $f^{*}$ is generally larger for big groups, individuals still change tasks less often as the variation of the labour distribution is generally lower (see figure 2).

So far, we have considered the responsiveness f as a fixed parameter in our simulations, arbitrarily controlling how individuals choose tasks as a function of their own proficiency and the distribution of labour at the group level. We now develop a fitness function to assess what would be the optimal value of f. If work done in one task was entirely substitutable with work done in another, fitness would be equal to the total work done and every individual should always pursue the task it is most competent at. Individuals would therefore never switch. In many cases, however, tasks should be somehow balanced within the group to enhance efficiency and manage indispensable duties. In the cooperatively breeding cichlid *N. pulcher*, for instance, a group that excavates additional breeding shelters might achieve a fitness benefit only if the additional shelters can also be sufficiently defended against predators of eggs and young. We therefore consider fitness to depend both on the total work done G and on the variation σ of the labour distribution across tasks:


(3.1) W=G−c⋅σ

where c is the fitness penalty imposed by variation of the labour distribution. We thus assume that as long as c>0, fitness is maximized if a given amount of work G is equally distributed between all tasks. With the simulation results from figure 2, we can now determine the value of *f* that maximizes group fitness W for a given value of c (figure 3). The higher the optimal responsiveness $f^{*}$, the more readily individuals switch between tasks (figure 1). Large groups have overall higher $f^{*}$ (figure 3A), but this does not lead to a weaker realized pattern of task specialization: individuals in small groups still change between tasks more often than individuals in large groups, due to the higher overall level of labour variation (figure 2B). Increasing the number of tasks reduces the optimal value of responsiveness (figure 3A) but increases the number of switches between tasks (figure 3B). As with group size, this is primarily due to the elevated labour variation found in groups that perform many tasks (figure 2B). The lower optimal responsiveness is additionally caused by a reduction of individuals’ prior experience in the tasks they perform when frequently switching between several instead of just two tasks. In small groups that perform three and five tasks, however, we observe that the number of task switches decreases as the number of tasks is increased. Small groups with an average group size of five frequently consist of exactly five individuals, which can more stably be distributed over five rather than three tasks. Such discrete numerical effects disappear at larger group sizes.

**Figure 4.**
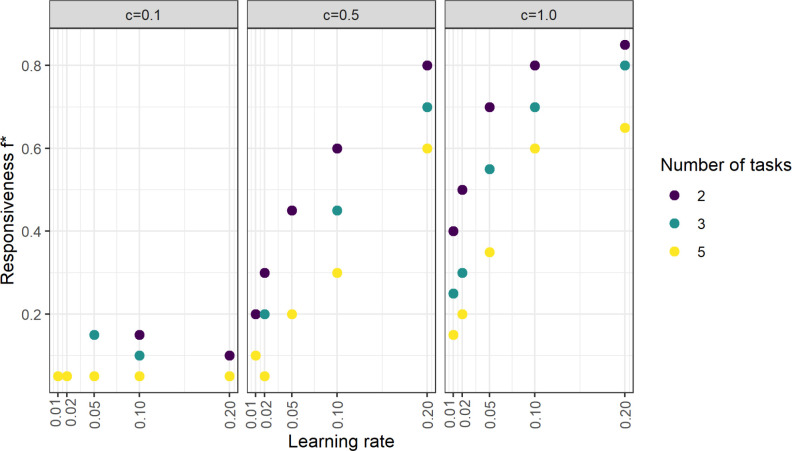
The optimal responsiveness depends on learning rate. The optimal responsiveness $f^{*}$ for a given variation penalty c increases as a function of learning rate. Simulations were performed for five different learning rates and responsiveness between 0 and 1 in 0.05 intervals, at an average group size of 10 (other parameters default to the standard values in table 1).

We also investigated the influence of learning rate on DoL. The optimal responsiveness $f^{*}$ increases with learning rate but decreases with the number of tasks (figure 4). Thus, individuals should switch between tasks more readily when they can learn a new task quickly and when they are more likely to switch back to a task for which they have previous experience.

For each optimal responsiveness $f^{*},$ we can determine the selection gradient with respect to learning and forgetting rates. Electronic supplementary material, figure S4 shows that a 10% increase in the learning rate leads to a fitness increase of around 0.2−0.6%, with an increasing trend from low to high values of $f^{*}$. The selection gradient on learning rates is higher overall in small groups than large groups, where task switching is generally less common. The selection gradient on learning rate also increases with the number of tasks the group needs to perform. Changes in the rate of forgetting, on the other hand, showed a much fainter selection gradient, where a 50% improvement (from 0.1 to 0.05) only yielded a maximum fitness increase of 0.2% at very high values for responsiveness, low learning rates and small group sizes (electronic supplementary material, figure S5).

## 4. Discussion

DoL, as measured by the (low) average number of task changes per lifetime, emerged under all parameter combinations of our model. As individuals are initially naive, they choose tasks based on the labour requirements of the group and therefore distribute themselves over the tasks, not unlike independent foragers that distribute themselves over a number of resource patches. The group may thereby arrive at a distribution at which no individual can reduce the variation of the labour distribution by changing its own task, similar to an ideal free distribution where no individual can improve its intake by switching between patches [40]. While individuals remain in a task, they gather competence through experience and thus become less likely to switch between tasks (figure 1B). The faster individuals learn, the quicker this process progresses and therefore we observe that high learning rates reduce the number of task switches for a given responsiveness.

As Ravary *et al*. [19] showed, individual experience can have a lasting effect on task choice and DoL. In our model, we observed that when individuals choose tasks based on previous experience, they readily distribute over tasks in such a way that task switches only rarely occur. But even when individual experience does not greatly inform task choice, the need to balance the group’s labour across tasks yields a distribution of individuals over tasks that leads to the emergence of task specialization.

The necessity to switch between tasks arises when additional individuals join the group, or established individuals die. New individuals are initially without experience, and this may lead to a situation where the established individuals remain in their dedicated tasks, while the newcomer starts switching between tasks to reduce inequalities in the labour distribution (figure 1B). A similar pattern has been observed in wood-eating termites that can switch between different castes during early developmental stages but later become specialized and lose this ability [41]. The developmental constraints that mark this transition in termites, however, are absent from our model; even old individuals can freely adopt any task and occasionally will do so. This particular form of temporal polyethism—where young, inexperienced individuals readily switch between tasks according to group needs, while older ones remain specialized—is something we expect to be particularly common in social vertebrates, where experience may play an important role in task performance. Empirical evidence is currently scarce. In cooperatively breeding cichlids, experiments have revealed that small (young) helpers respond more flexibly to territory intrusions than larger (older) helpers by adjusting their threat behaviour and shelter maintenance to the actual type of challenge [42], and task performance of helpers is influenced by their age (size) and preferred location in the territory [18]. In humans, a decrease in job-switching frequency with age is well documented ([43] and citations therein).

What are the effects of group size on the processes described above? First of all, a larger group size under the assumption of a constant mortality rate of its members reduces the amount of demographic stochasticity (electronic supplementary material, figure S1). A single mortality event is more impactful on the distribution of labour in a group of 5 than in a group of 33. Thus, the labour variation is generally higher in small than in large groups (figure 2B), and to balance the labour distribution individuals start switching sooner (electronic supplementary material, figure S6) and lose more task competence (figure 2A) in small than in large groups. Only at high responsiveness and a larger number of tasks, this group-size effect may change to the opposite, where frequent small-effect changes of the group composition induce more task switching in large groups (figure 2A, middle and right panel). These findings do not, however, indicate that individuals in large groups have a stronger evolved tendency for task specialization. Indeed, the evolutionary optima for large groups yield a higher responsiveness to group requirements. Due to the lower overall labour variation, however, this results in a higher *realized* task specialization.

In our simulations, the number of tasks is generally lower than the number of individuals. If the number of tasks exceeds the number of individuals, some tasks would not be fulfilled, which implies that task switching would increase. If responsiveness is sufficiently low, however, individuals might still specialize if the benefit gained from their experience outweighs the disadvantage caused by an imbalance in labour distribution. Modelling a scenario where some tasks are left unattended due to group size limitations should consider also for how long the effects of task performance will prevail (see e.g. [44]).

The influence of group size on DoL has been studied empirically in a number of species [26–29,45–47], but the mechanisms underlying group-size effects are largely unclear. Pharaoh’s ants *Monomorium pharaonis* show a transition from unorganized to organized foraging behaviour above a colony size of 500 individuals, which was hypothesized to be due to the volatility of trail pheromones and the associated inability of small colonies to form stable trails [46]. Such effects of scale may be important in the evolution of colony size, but this does not relate to the behavioural mechanisms scrutinized in our model. Wasps show frequent transitions between pulp foraging, water foraging and building in small colonies, and an increasing tendency of task specialization in large colonies [48]. In our model, the different tasks are not explicitly integrated with each other (task partitioning, [49]), but in wasps, the need to balance these three tasks for the group to succeed may resemble the situation in our model in which the fitness cost of labour variation is high. The mechanism by which specialization arose in wasp societies is unclear [48].

Theoretical models have investigated group-size effects from various angles, including threshold reinforcement [31], fixed response thresholds [29,32], interaction effects [30] and task partitioning [49]. All these models have in common that the effect of group size on DoL is positive. The larger number of individuals in response-threshold models [29,31,32] reduces the variation in stimulus levels that triggers individual activity. Thus, only a few individuals repeatedly pursue a task, either due to innate variation [29,32] or reinforcement [31]. Although the assumptions of our model differ in important respects (individuals continuously perform tasks, improve their task performance through experience and are sensitive to their own task competence), we recover similar results in the form of a reduction of labour variation in large groups that leads to a decreased number of task switches and therefore increased DoL. An important difference, however, is that the effect on DoL in the other models is derived from the group size itself, whereas in our model it arises through the increased demographic stochasticity characterizing small groups; if the group composition were stable over the long run, task specialization and DoL would emerge regardless of group size.

Anderson & Ratnieks [49] considered the effect of group size in a task-partitioning model, where group members together perform a number of functionally integrated tasks. The larger number of individuals leads to a smoother transition between sequential tasks and reduces queuing delay (for instance when a wasp that foraged for pulp waits to transfer material to a nest-mate; [48]). Their model presupposes a regime of DoL that gains efficiency through increased group size. The effect of queuing delays is related to variation in the degree to which certain tasks are performed by the group, and therefore represents a situation where, in terms of our model, variation of the labour distribution is strongly penalized. We found that for a given variation penalty, the responsiveness to switch between tasks increases with group size, but that the overall number of switches decreases.

Concerning the effect of learning rates on DoL, we found that the learning rate is under stronger selection in small than in large groups. This is because individuals in small groups are more often forced to switch between tasks and therefore regularly perform tasks for which they have no previous experience. The rate of learning thus has a stronger influence on fitness in small groups where individuals have to learn and relearn tasks more often than in large groups where individuals have a higher level of task specialization. One might therefore suggest that all else being equal, DoL and its coordination are cognitively more demanding in smaller groups, in contrast to the social brain hypothesis that predicts larger group sizes to increase cognitive demands and brain sizes [50]. Our model, however, only considers the DoL aspect of group living, not the role of conflict and social interactions.

A key limitation of our modelling approach is that we disregarded potential conflicts of interest between group members by assuming a complete alignment between individual and group fitness, suggesting that group-level adaptation may prevent intra-group conflict [51]. This might be a reasonable assumption if all tasks are equally costly to perform, and there is no cost to individual fitness in switching between tasks. Our study cannot provide insights, however, into how group size may affect DoL via the negotiation of conflicts between individual and collective interests, which is a drawback as the conflict potential may be particularly prevalent in smaller social groups below the threshold of eusocial colonies. Future work should therefore address the role of negotiation and conflict resolution on the emergence of DoL in groups of varying sizes.

## Data Availability

Simulation model code and data analysis are available on Zenodo [52]. Supplementary material is available online [53].

## References

[1] Wilson EO. 1971 The insect societies. Cambridge, MA: Belknap Press.

[2] Brahma A, Mandal S, Gadagkar R. 2018 Emergence of cooperation and division of labor in the primitively eusocial wasp Ropalidia marginata. Proc. Natl Acad. Sci. **115**, 756–761. (10.1073/pnas.1714006115) 29311307 PMC5789922

[3] Gazda SK, Connor RC, Edgar RK, Cox F. 2005 A division ohttps://doi.org/10.6084/m9.figshare.c.7690686f labour with role specialization in group–hunting bottlenose dolphins (Tursiops truncatus) off Cedar Key, Florida. Proc. R. Soc. B **272**, 135–140. (10.1098/rspb.2004.2937) PMC1634948 15695203

[4] Serra J, Hurtado MJ, Le Négrate A, Féron C, Nowak R, Gouat P. 2012 Behavioral differentiation during collective building in wild mice Mus spicilegus. Behav. Process. **89**, 292–298. (10.1016/j.beproc.2011.12.007) 22206995

[5] Settepani V, Grinsted L, Granfeldt J, Jensen JL, Bilde T. 2013 Task specialization in two social spiders, Stegodyphus sarasinorum (Eresidae) and Anelosimus eximius (Theridiidae). J. Evol. Biol. **26**, 51–62. (10.1111/jeb.12024) 23163349

[6] Zhang Z, Claessen D, Rozen DE. 2016 Understanding microbial divisions of labor. Front. Microbiol. **7**, 235404. (10.3389/fmicb.2016.02070) PMC5174093 28066387

[7] West SA, Cooper GA. 2016 Division of labour in microorganisms: an evolutionary perspective. Nat. Rev. Microbiol. **14**, 716–723. (10.1038/nrmicro.2016.111) 27640757

[8] Smith MG, Riehl C. 2022 Workload distribution and division of labor in cooperative societies. Q. Rev. Biol. **97**, 183–210. (10.1086/721520)

[9] Taborsky M. 2025 The evolution of division of labour: preconditions and evolutionary feedback. Phil. Trans. R. Soc. B **380**, 20230262. (10.1098/rstb.2023.0262) 40109117 PMC11923618

[10] Smith A. 1776 An inquiry into the nature and causes of the wealth of nations. London, UK: Straman & Cadfli.

[11] Durkheim É. 1893 De la division du travail social. Paris, UK: Presses Universitaires de France.

[12] Ispolatov I, Ackermann M, Doebeli M. 2012 Division of labour and the evolution of multicellularity. Proc. R. Soc. B **279**, 1768–1776. (10.1098/rspb.2011.1999) PMC3297448 22158952

[13] Cooper GA, West SA. 2018 Division of labour and the evolution of extreme specialization. Nat. Ecol. Evol. **2**, 1161–1167. (10.1038/s41559-018-0564-9) 29807994

[14] Oster GF, Wilson EO. 1978 Caste and ecology in the social insects. Princeton, NJ: Princeton University Press. 740003

[15] Mertl AL, Traniello JFA. 2009 Behavioral evolution in the major worker subcaste of twig-nesting Pheidole (Hymenoptera: Formicidae): does morphological specialization influence task plasticity? Behav. Ecol. Sociobiol. **63**, 1411–1426. (10.1007/s00265-009-0797-3)

[16] Ito K, Higginson A. 2025 Specialism and generalism in social animals in variable environments. Phil. Trans. R. Soc. B **380**, 20230264. (10.1098/rstb.2023.0264) 40109114 PMC11923619

[17] Robinson G. 1992 Regulation of division of labor in insect societies. Annu. Rev. Entomol. **37**, 637–665. (10.1146/annurev.ento.37.1.637) 1539941

[18] Bruintjes R, Taborsky M. 2011 Size-dependent task specialization in a cooperative cichlid in response to experimental variation of demand. Anim. Behav. **81**, 387–394. (10.1016/j.anbehav.2010.10.004)

[19] Ravary F, Lecoutey E, Kaminski G, Châline N, Jaisson P. 2007 Individual experience alone can generate lasting division of labor in ants. Curr. Biol. **17**, 1308–1312. (10.1016/j.cub.2007.06.047) 17629482

[20] Chittka L, Muller H. 2009 Learning, specialization, efficiency and task allocation in social insects. Commun. Integr. Biol **2**, 151–154. (10.4161/cib.7600) 19513269 PMC2686371

[21] Fewell JH, Page RE. 1999 The emergence of division of labour in forced associations of normally solitary ant queens. Evol. Ecol. Res **1**, 537–548.

[22] Tate Holbrook C, Clark RM, Jeanson R, Bertram SM, Kukuk PF, Fewell JH. 2009 Emergence and consequences of division of labor in associations of normally solitary sweat bees. Ethology **115**, 301–310. (10.1111/j.1439-0310.2009.01617.x)

[23] Chittka L. 2017 Bee cognition. Curr. Biol. **27**, R1049–R1053. (10.1016/j.cub.2017.08.008) 29017035

[24] Bridges AD, Royka A, Wilson T, Lockwood C, Richter J, Juusola M, Chittka L. 2024 Bumblebees socially learn behaviour too complex to innovate alone. Nature **627**, 572–578. (10.1038/s41586-024-07126-4) 38448580 PMC10954542

[25] Bell-Roberts L, Turner JFR, Werner GDA, Downing PA, Ross L, West SA. 2024 Larger colony sizes favoured the evolution of more worker castes in ants. Nat. Ecol. Evol. **8**, 1959–1971. (10.1038/s41559-024-02512-7) 39187609 PMC7616618

[26] Ferguson-Gow H, Sumner S, Bourke AFG, Jones KE. 2014 Colony size predicts division of labour in attine ants. Proc. R. Soc. B **281**, 20141411. (10.1098/rspb.2014.1411) PMC4173680 25165765

[27] Holbrook CT, Kukuk PF, Fewell JH. 2013 Increased group size promotes task specialization in a normally solitary halictine bee. Behaviour **150**, 1449–1466. (10.1163/1568539x-00003104)

[28] Amador-Vargas S, Gronenberg W, Wcislo WT, Mueller U. 2015 Specialization and group size: brain and behavioural correlates of colony size in ants lacking morphological castes. Proc. R. Soc. B **282**, 20142502. (10.1098/rspb.2014.2502) PMC4309001 25567649

[29] Ulrich Y, Saragosti J, Tokita CK, Tarnita CE, Kronauer DJC. 2018 Fitness benefits and emergent division of labour at the onset of group living. Nature **560**, 635–638. (10.1038/s41586-018-0422-6) 30135576 PMC6121774

[30] Pacala SW, Gordon DM, Godfray HCJ. 1996 Effects of social group size on information transfer and task allocation. Evol. Ecol. **10**, 127–165. (10.1007/bf01241782)

[31] Gautrais J, Theraulaz G, Deneubourg JL, Anderson C. 2002 Emergent polyethism as a consequence of increased colony size in insect societies. J. Theor. Biol. **215**, 363–373. (10.1006/jtbi.2001.2506) 12054843

[32] Jeanson R, Fewell JH, Gorelick R, Bertram SM. 2007 Emergence of increased division of labor as a function of group size. Behav. Ecol. Sociobiol. **62**, 289–298. (10.1007/s00265-007-0464-5)

[33] Nakahashi W, Feldman MW. 2014 Evolution of division of labor: emergence of different activities among group members. J. Theor. Biol. **348**, 65–79. (10.1016/j.jtbi.2014.01.027) 24486228

[34] Bonabeau E. 1998 Fixed response thresholds and the regulation of division of labor in insect societies. Bull. Math. Biol. **60**, 753–807. (10.1006/bulm.1998.0041)

[35] Theraulaz G, Bonabeau E, Denuebourg JN. 1998 Response threshold reinforcements and division of labour in insect societies. Proc. R. Soc. Lond. B **265**, 327–332. (10.1098/rspb.1998.0299)

[36] Duarte A, Pen I, Keller L, Weissing FJ. 2012 Evolution of self-organized division of labor in a response threshold model. Behav. Ecol. Sociobiol. **66**, 947–957. (10.1007/s00265-012-1343-2) 22661824 PMC3353103

[37] Jeanson R, Weidenmüller A. 2014 Interindividual variability in social insects: proximate causes and ultimate consequences. Biol. Rev. **89**, 671–687. (10.1111/brv.12074) 24341677

[38] Jeanson R. 2019 Within-individual behavioural variability and division of labour in social insects. J. Exp. Biol. **222**, b190868. (10.1242/jeb.190868) 31127006

[39] Beshers SN, Fewell JH. 2001 Models of division of labor in social insects. Annu. Rev. Entomol. **46**, 413–440. (10.1146/annurev.ento.46.1.413) 11112175

[40] Fretwell SD, Lucas HL. 1969 On territorial behavior and other factors influencing habitat distribution in birds. Acta Biotheor. **19**, 70. (10.1007/bf01601953)

[41] Korb J, Hartfelder K. 2008 Life history and development: a framework for understanding developmental plasticity in lower termites. Biol. Rev. **83**, 295–313. (10.1111/j.1469-185x.2008.00044.x) 18979593

[42] Taborsky M, Hert E, Siemens M, Stoerig P. 1986 Social behaviour of Lamprologus species: functions and mechanisms. Annales du Musée Royale de l’Afrique Central, Serie Zoologie **251**, 7–11.

[43] Carless SA, Arnup JL. 2011 A longitudinal study of the determinants and outcomes of career change. J. Vocat. Behav. **78**, 80–91. (10.1016/j.jvb.2010.09.002)

[44] Staps M, Tarnita CE. 2022 When being flexible matters: Ecological underpinnings for the evolution of collective flexibility and task allocation. Proc. Natl Acad. Sci. **119** 1-12. (10.1073/pnas.2116066119) PMC9170069 35486699

[45] Balshine S, Leach B, Neat F, Reid H, Taborsky M, Werner N. 2001 Correlates of group size in a cooperatively breeding cichlid fish (Neolamprologus pulcher). Behav. Ecol. Sociobiol. **50**, 134–140. (10.1007/s002650100343)

[46] Beekman M, Sumpter DJT, Ratnieks FLW. 2001 Phase transition between disordered and ordered foraging in Pharaoh’s ants. Proc. Natl Acad. Sci. **98**, 9703–9706. (10.1073/pnas.161285298) 11493681 PMC55516

[47] Fischer S, Zöttl M, Groenewoud F, Taborsky B. 2014 Group-size-dependent punishment of idle subordinates in a cooperative breeder where helpers pay to stay. Proc. R. Soc. B **281**, 20140184. (10.1098/rspb.2014.0184) PMC4100499 24990673

[48] Karsai I, Wenzel JW. 1998 Productivity, individual-level and colony-level flexibility, and organization of work as consequences of colony size. Proc. Natl. Acad. Sci. **95**, 8665–8669. (10.1073/pnas.95.15.8665) 9671735 PMC21133

[49] Anderson C, Ratnieks FLW. 1999 Task partitioning in insect societies. I. Effect of colony size on queueing delay and colony ergonomic efficiency. Am. Nat. **154**, 521–535. (10.1086/303255) 10561125

[50] Dunbar RIM. 1998 The social brain hypothesis. Evol. Anthropol. **6**, 178–190. (10.1002/(sici)1520-6505(1998)6:53.3.co;2-p)

[51] Korb J, Heinze J. 2004 Multilevel selection and social evolution of insect societies. Naturwissenschaften **91**, 291–304. (10.1007/s00114-004-0529-5) 15241605

[52] Netz C. 2025 DoL simulation. (10.5281/zenodo.14679143)

[53] Netz C, Fawcett T, Higginson A, Taborsky M, Taborsky B. 2025 Supplementary material from: Group size and labour demands determine division oflabour as a consequence of demographic stochasticity. Figshare (10.6084/m9.figshare.c.7690686) PMC11923606 40109102

